# Supplementary material for: A local ATR-dependent checkpoint pathway is activated by a site-specific replication fork block in human cells
Source: bioRxiv. 2023 Mar 26:2023.03.26.534293. Preprint. [Version 1] doi: 10.1101/2023.03.26.534293 (PMC10055377; doi:10.1101/2023.03.26.534293)
Supplement: 1 [file NIHPP2023.03.26.534293v1-supplement-1.pdf]

bioRxiv preprint doi: https://doi.org/10.1101/2023.03.26.534293; this version posted March 26, 2023. The copyright holder for this preprint (which was not certified by peer review) is the author/funder, who has granted bioRxiv a license to display the preprint in perpetuity. It is made available under aCC-BY 4.0 International license.

777 **Supplementary Figures**

Sup Figure 1:

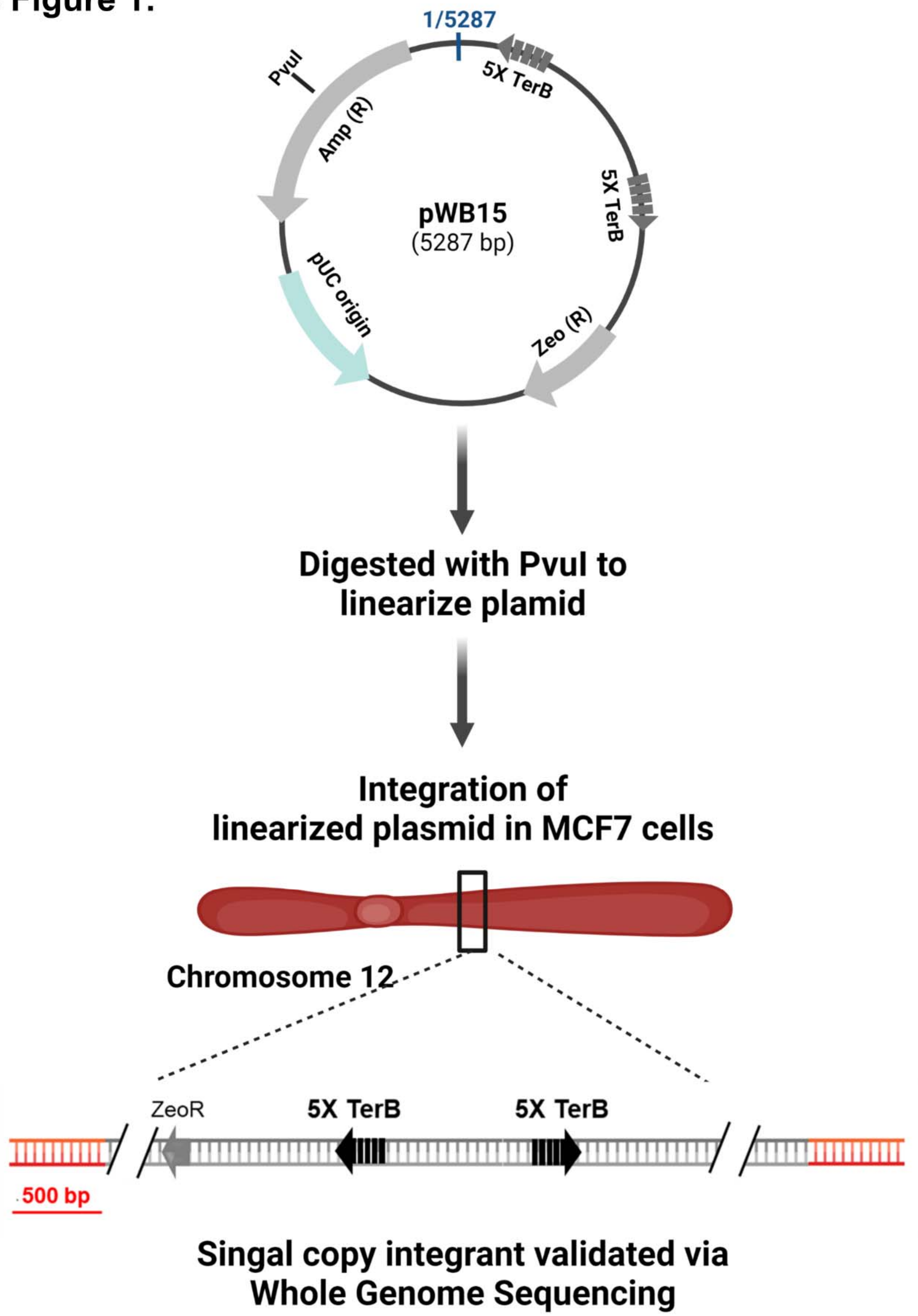

778

bioRxiv preprint doi: https://doi.org/10.1101/2023.03.26.534293; this version posted March 26, 2023. The copyright holder for this preprint (which was not certified by peer review) is the author/funder, who has granted bioRxiv a license to display the preprint in perpetuity. It is made available under aCC-BY 4.0 International license.

Supplementary Figure 1. Generation of the MCF7 5C-TerB cell line: Schematic of the integration of pWB15 in MCF7 cells to generate MCF7 5C-TerB. pWB15 contains two *TerB* cassettes (grey arrows). Each *TerB* cassette contains 5 tandem *TerB* sequences, which are in the non-permissive orientation in pWB15 (grey arrow facing away from each other. The plasmid was linearized by digesting with PvuI for integration into MCF7 cells. The single copy integrant was confirmed using whole genome sequencing and found to be integrated at Chromosome 12.

bioRxiv preprint doi: https://doi.org/10.1101/2023.03.26.534293; this version posted March 26, 2023. The copyright holder for this preprint (which was not certified by peer review) is the author/funder, who has granted bioRxiv a license to display the preprint in perpetuity. It is made available under aCC-BY 4.0 International license.

Sup Figure 2:

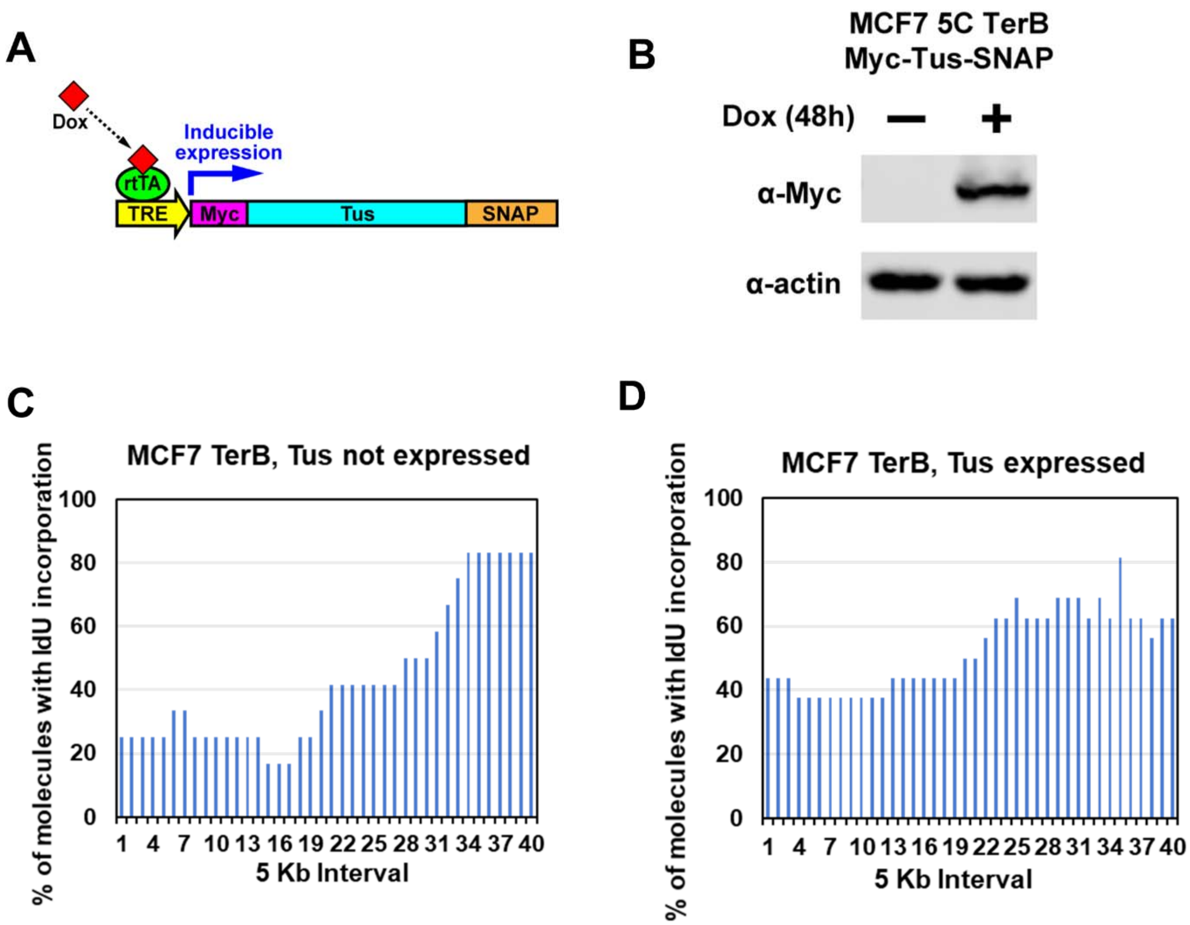

Supplementary Figure 2. **Expression of Tus in MCF7 5C-TerB cells: (A)** Schematic of Doxycycline (Dox) inducible expression of Myc-NLS-TUS-SNAP. (rtTA = reverse tetracycline trans activator; TRE = tetracycline response element). **(B)** Immunoblot of MCF7 5C-TerB cells stably expressing Dox inducible Myc-NLS-TUS-SNAP. **(C-D)** Replication profiles shown as the percentage of molecules with IdU incorporation at each 5 Kb interval in the 200 Kb SfiI segment containing *TerB* sequence, quantified from molecules MCF7 5C-TerB in Figure 2C (Tus not expressed) and Figure 2D (Tus expressed).

bioRxiv preprint doi: https://doi.org/10.1101/2023.03.26.534293; this version posted March 26, 2023. The copyright holder for this preprint (which was not certified by peer review) is the author/funder, who has granted bioRxiv a license to display the preprint in perpetuity. It is made available under aCC-BY 4.0 International license.

Sup Figure 3:

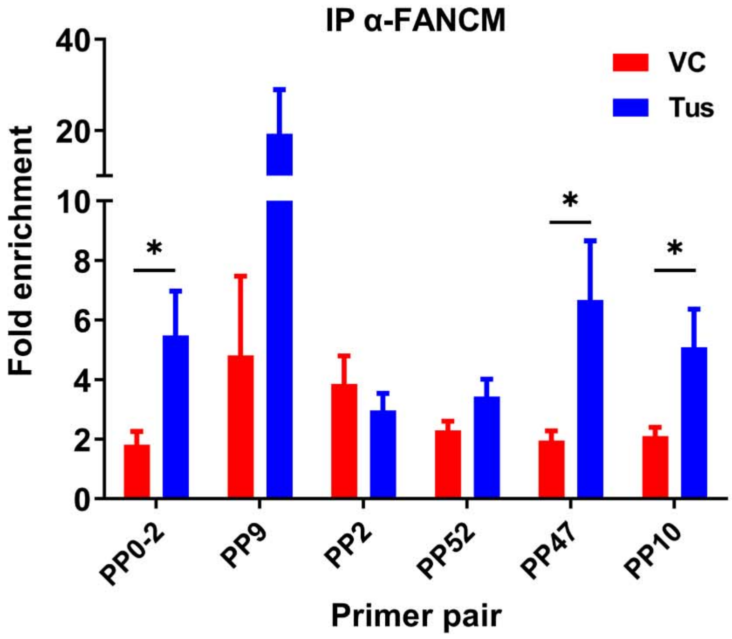

Supplementary Figure 3. Enrichment of FANCM at the Ter sequence in the presence of the Tus protein: ChIP using FANCM antibody was performed MCF7 5C-TerB cells transfected with VC or HA-Tus-His plasmids. ChIP-qPCR were conducted using the indicated primer pairs. Data shows the fold enrichment relative to the IgG controls (n=3).

bioRxiv preprint doi: https://doi.org/10.1101/2023.03.26.534293; this version posted March 26, 2023. The copyright holder for this preprint (which was not certified by peer review) is the author/funder, who has granted bioRxiv a license to display the preprint in perpetuity. It is made available under aCC-BY 4.0 International license.

Sup Figure 4.

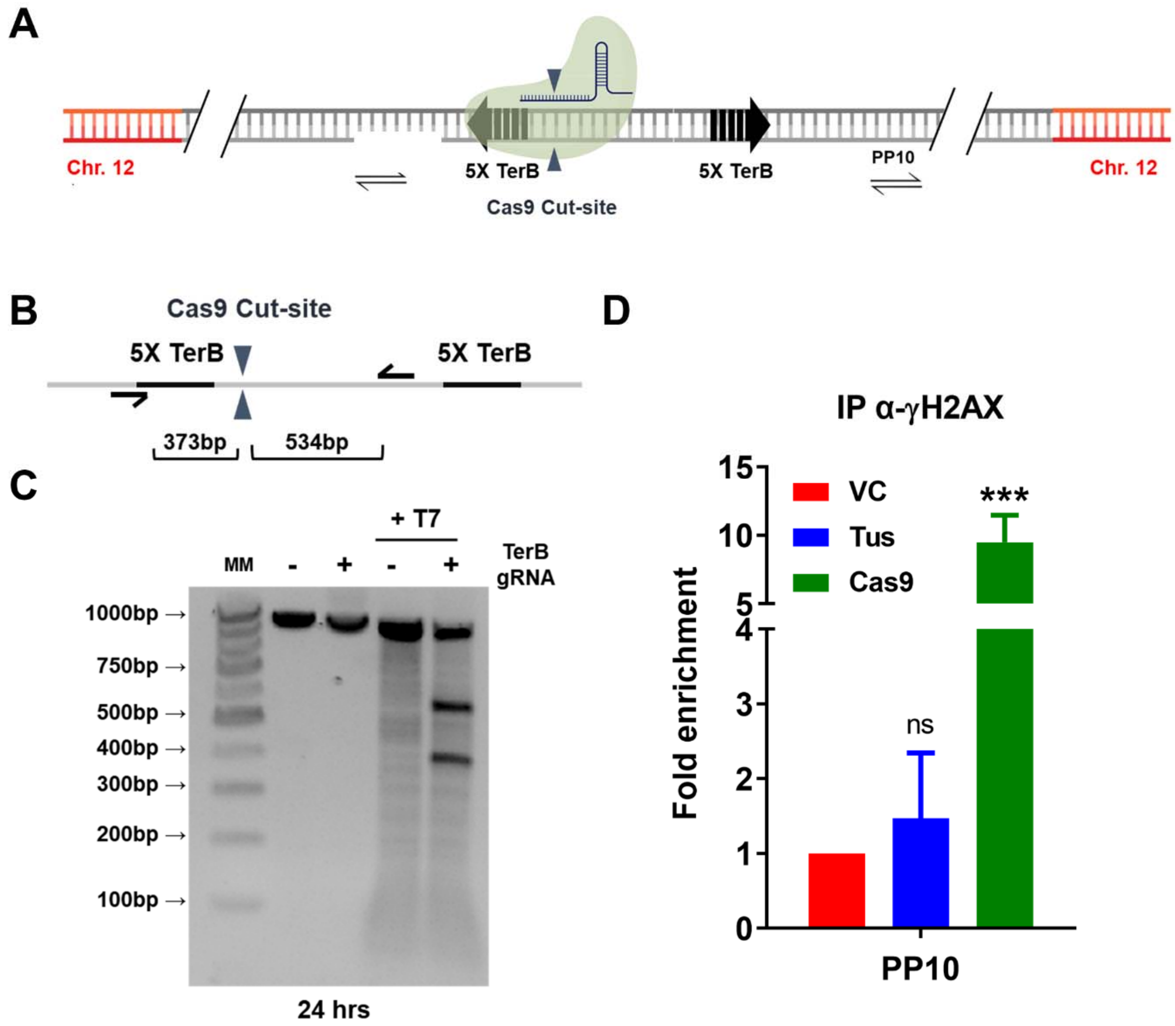

Supplementary Figure 4. Distinct patterns of γH2AX enrichment at a Cas9-mediated DSB vs the Tus-*TerB* fork barrier: (A) Schematic of the linearized *TerB* plasmid (pWB15) integrated as a unique copy into MCF7 cells (MCF7 5C-TerB) with the Cas9-binding site (green protein). Blue triangles: Cas9 cut site. Black half-arrow heads depict PCR products expected from primer pair (PP10) used in quantitative PCR (qPCR) are shown. **(B)** Schematic depicting the Cas9 cut site (blue arrows), site-specific PCR primers (black half-arrowhead) and predicted size of cleavage products. **(C)** PCR analysis by T7 assay of MCF7 5C-TerB cells transfected with Cas9-sgRNA RNP complex. **(D)** γH2AX levels along the integrated *TerB* plasmid were analyzed by ChIP-qPCR in MCF7 5C-TerB cells transfected with VC, Tus or Cas9 expression

bioRxiv preprint doi: https://doi.org/10.1101/2023.03.26.534293; this version posted March 26, 2023. The copyright holder for this preprint (which was not certified by peer review) is the author/funder, who has granted bioRxiv a license to display the preprint in perpetuity. It is made available under aCC-BY 4.0 International license.

plasmids using the indicated primer pair. Data shows the fold enrichment relative to IgG controls (n=2).

bioRxiv preprint doi: https://doi.org/10.1101/2023.03.26.534293; this version posted March 26, 2023. The copyright holder for this preprint (which was not certified by peer review) is the author/funder, who has granted bioRxiv a license to display the preprint in perpetuity. It is made available under aCC-BY 4.0 International license.

Sup Figure 5.

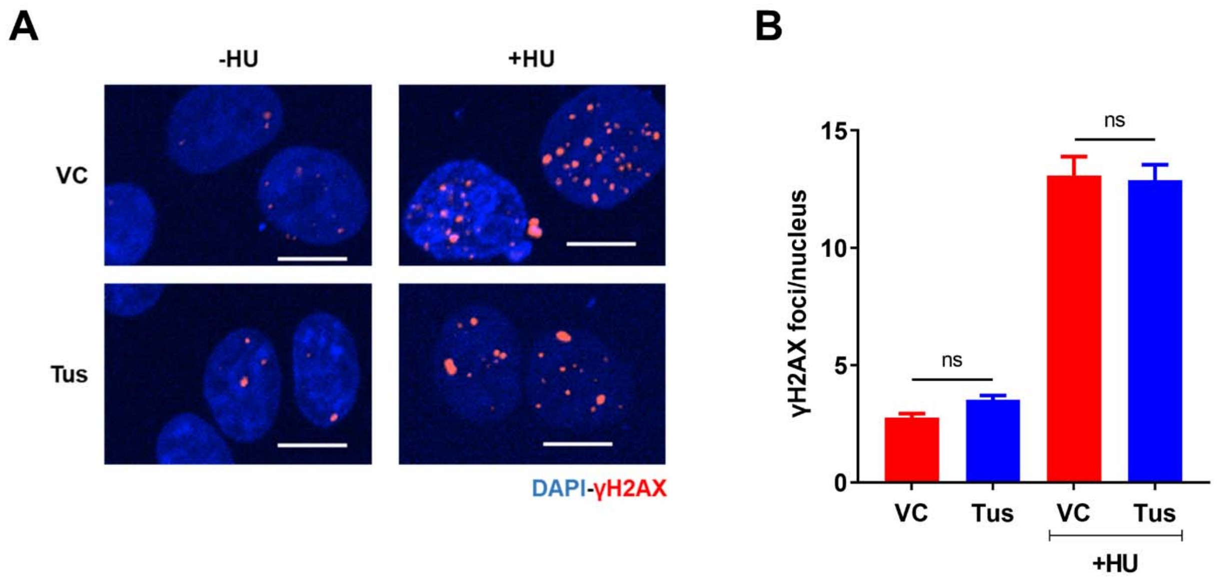

Supplementary Figure 5. Genome-wide gH2AX foci were unaffected with Tus expression. (A) Representative images of gH2AX foci across stated conditions. Bars, 10 µm **(B)** Average number of gH2AX foci per nucleus in conditions stated. Cells treated with or without 2mM HU for 4 hours. (n=3, ≥300 cells per experiment)

bioRxiv preprint doi: https://doi.org/10.1101/2023.03.26.534293; this version posted March 26, 2023. The copyright holder for this preprint (which was not certified by peer review) is the author/funder, who has granted bioRxiv a license to display the preprint in perpetuity. It is made available under aCC-BY 4.0 International license.

Sup Figure 6.

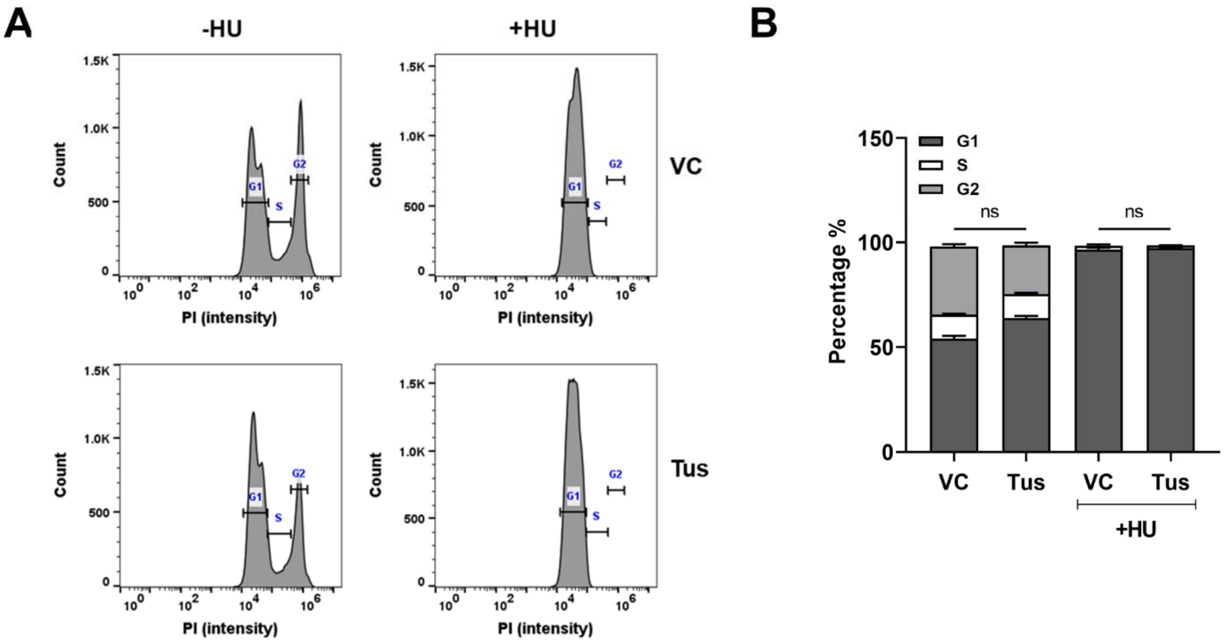

824

825 **Supplementary Figure 6. Cell cycle progression were unaffected with Tus**
826 **expression. (A)** Cell cycle analysis and **(B)** quantification was performed using flow
827 cytometry following staining with propidium iodide staining in MCF7 5C-TerB cells under
828 the stated conditions. Cells treated with or without 2mM HU for 4 hours. (n=3, ns: non-
829 significant)

830

bioRxiv preprint doi: https://doi.org/10.1101/2023.03.26.534293; this version posted March 26, 2023. The copyright holder for this preprint (which was not certified by peer review) is the author/funder, who has granted bioRxiv a license to display the preprint in perpetuity. It is made available under aCC-BY 4.0 International license.

Supplementary Tables

Supplementary Table 1. Demonstration that the Tus-Ter replication fork block does not activate significant replication elsewhere in the genome. Replication characteristics of 200 kb global DNA segments that represent the total genome. These measurements do not include the segments containing the *TerB* sequence. The table compares MCF7 cells containing the *TerB* sequence with and without Tus induced. This was determined on stretched DNA molecules that had completely incorporated IdU, CldU or a combination of both nucleotide analogues.

Supplementary Table 2. List of antibodies, oligos and plasmids used in the study.
